# Safe start at home: what parents of newborns need after early discharge from hospital – a focus group study

**DOI:** 10.1186/s12913-016-1300-2

**Published:** 2016-03-08

**Authors:** Elisabeth Kurth, Katrin Krähenbühl, Manuela Eicher, Susanne Rodmann, Luzia Fölmli, Cornelia Conzelmann, Elisabeth Zemp

**Affiliations:** Institute of Midwifery, Zurich University of Applied Sciences, Winterthur, Switzerland; Swiss Tropical and Public Health Institute, Basel, Switzerland; University of Basel, Basel, Switzerland; Midwifery-Network, Familystart beider Basel, Basel, Switzerland; Health Division, Bern University of Applied Sciences, Bern, Switzerland; School of Health Sciences Fribourg, University of Applied Arts and Sciences Western Switzerland, Fribourg, Switzerland; Institute of Higher Education and Research in Healthcare, University of Lausanne, Lausanne, Switzerland; Department of Obstetrics and Gynecology, University Hospital, Basel, Switzerland; Parents Counselling Basel-Stadt, Basel, Switzerland; UNICEF Schweiz, Zürich, Switzerland

**Keywords:** Postnatal care, Patient satisfaction, Interprofessional collaboration, Length of hospital stay, Health visiting, Midwifery

## Abstract

**Background:**

The length of postpartum hospital stay is decreasing internationally. Earlier hospital discharge of mothers and newborns decreases postnatal care or transfers it to the outpatient setting. This study aimed to investigate the experiences of new parents and examine their views on care following early hospital discharge.

**Methods:**

Six focus group discussions with new parents (*n* = 24) were conducted. A stratified sampling scheme of German and Turkish-speaking groups was employed. A ‘playful design’ method was used to facilitate participants communication wherein they used blocks and figurines to visualize their perspectives on care models The visualized constructions of care models were photographed and discussions were audio-recorded and transcribed verbatim. Text and visual data was thematically analyzed by a multi-professional group and findings were validated by the focus group participants.

**Results:**

Following discharge, mothers reported feeling physically strained during recuperating from birth and initiating breastfeeding. The combined requirements of infant and self-care needs resulted in a significant need for practical and medical support.

Families reported challenges in accessing postnatal care services and lacking inter-professional coordination. The visualized models of ideal care comprised access to a package of postnatal care including monitoring, treating and caring for the health of the mother and newborn. This included home visits from qualified midwives, access to a 24-h helpline, and domestic support for household tasks. Participants suggested that improving inter-professional networks, implementing supervisors or a centralized coordinating center could help to remedy the current fragmented care.

**Conclusions:**

After hospital discharge, new parents need practical support, monitoring and care. Such support is important for the health and wellbeing of the mother and child. Integrated care services including professional home visits and a 24-hour help line may help meet the needs of new families.

**Electronic supplementary material:**

The online version of this article (doi:10.1186/s12913-016-1300-2) contains supplementary material, which is available to authorized users.

## Background

Internationally, there is a growing trend towards earlier hospital discharge after birth [1]. In Switzerland, average postnatal hospital stays have been more than halved over the last 50 years - from nearly 2 weeks to 5 days [2, 3]. Most recently, the introduction of diagnosis-related group (DRG) reimbursement has resulted in even shorter stays of 3 days after spontaneous vaginal birth [4]. These reductions limit the time available for monitoring maternal and child health as well as for parenting education by health professionals.

The definition of early discharge varies widely, and ranges from 6 to 72 h post-delivery [5]. The impact of early postnatal discharge policies have been predominantly examined in western, English-speaking countries. Epidemiologic and observational studies have shown that early discharge (i.e. < 30 h post-birth) is associated with increased risk of newborn hospital readmission (e.g. jaundice, dehydration, and sepsis) and preventable neonatal mortality (e.g. cardiac problems, infection) [6–8]. However, a recent Cochrane review did not find statistically significant differences in infant or maternal readmissions or mortality [5]. Yet, the power of the reviewed RCT-studies’ pooled results did not allow valid results on these rare outcomes. Importantly, there was clear evidence that early discharge had no adverse effects on maternal depression (or breastfeeding rates) when families were offered at least one professional home visit post-discharge [5]. Further, several follow-up transitional care programs including home visits by a midwife or a public health nurse have demonstrated improved maternal confidence, more successful breastfeeding and decreased postpartum fatigue and depression [9–11]. However, not all home-visit programs are universally successful [12]. Other postnatal care interventions such as providing written information, group meetings or early outpateint follow-up visits have not shown positive effects on maternal/child health outcomes [13, 14]. Similarly, on-demand home visits or at-home consultations to address complications have not clearly demonstrated positive effects on health outcomes [15]. Telehealth interventions including phone-based peer-support programs to support breastfeeding and address postpartum depression have been quite effective [16, 17]. An extended nurse telephone intervention was associated with lower perceived stress and lower infant health care charges [18].

To date, new parents’ desires for postnatal care services after early discharge have only been addressed in a few studies. When given the option, most new mothers prefer a longer hospital stay or a period in a postnatal hotel [19, 20]. A commonly reported reason for this is the perceived need for 24-hour professional surveillance and support [20, 21]. Two Swedish studies found that around-the-clock (24 h) access to health-care professionals was also a frequent wish of new parents facing early discharge [22, 23].

### Post-discharge care provision in Switzerland

In Switzerland, new mothers can receive home visits from an independent midwife up to 10 days postpartum and this can be extended by medical prescription. These visits are reimbursed by the mandatory health insurance program. In most regions, the parents are responsible for finding a midwife. However, several factors including, language and cultural barriers (i.e. immigrants) combined with a lack of midwives in some regions means that such in-home visits are not guaranteed. In community-based facilities, health visitors offer consultation-hours providing counseling on infant care and parenting. In some regions, health visitors can offer home visits if necessary. Typically, mothers receive a 6-week postnatal follow-up by an obstetrician, while the newborn is scheduled for initial visit and vaccinations with a pediatrician. However, these ambulatory visits are conducted independently, and as such, postnatal care is highly fragmented with a lack of coordination across hospital inpatient, ambulatory, and community-based settings [24].

When the introduction of the DRG reimbursement system was planned for 2012, many health experts expected further reductions in hospital stays (from 5 to 3 days) and a resulting reduction or transfer of care to the outpatient setting. To fill the emerging gap, a group of independent midwives working in the Swiss canton of Basel sought to design a coordinated, needs-oriented postnatal care service for families following hospital discharge. This needs assessment study aimed to investigate new parents’ perspectives and experiences with professional care during the initial transition period with the newborn at home. Further we sought to gather their views on the content and organization of post-discharge care services after an early hospital discharge (< 72 h after birth).

## Methods

### Design

We used a qualitative research design to understand new parents’ experiences and expectations of postnatal care after hospital discharge [25]. Focus group discussions were employed as social interactions within a group can help facilitate and lead to an in-depth exploration of emerging themes raised by participants and at the same time is a means to involve users in evaluating and developing health care services [26, 27]. In parallel, we incorporated a ‘playful design’ approach [28] previously used in organizational development and strategic planning [29]. Visualizing through metaphors and models can lead to a shared understanding of a situation and to strategies for action [29]. Additionally, enabling participants to manually express themselves using building bricks and figurines (toys) to visualize a personal viewpoint or experience may be useful for encouraging verbally weak participants to become actively involved in the group discussion.

### Participants and sampling

The study was conducted in the region of Basel, Switzerland, which encompasses rural, suburban and urban areas with a large immigrant population. Adults who became parents within the last 9 months (prior to data collection) and who could speak Swiss German, German or Turkish were invited to participate. The time frame was selected to ensure that participants had enough time to use different postnatal care services yet short enough so that they were still able to recall their experiences. To account for the relatively high proportion of immigrant parents, who might face different challenges related to information, communication and access to care, we included Turkish mothers, which is among the largest of immigrant populations in the region. In total, 6 focus groups were planned, with a target of 4–6 participants in each to facilitate active involvement in the ‘playful design’ component. Five focus groups were to be held for women (four German speaking, one Turkish speaking). As fathers are often excluded from studies on postnatal care, we planned a single group specifically for new fathers (German speaking). To include participants with a broad range of diverse backgrounds we used a stratified sampling, accounting for gender and language, parity, degree of education and for urban or suburban/rural residence. To ensure that participants reflected experiences with different outpatient postpartum care services, we engaged regional midwife, health visitor, gynecologic and pediatric associations to support participant recruitment. The members of these associations were informed about the study by mailing or personal contact and if they were willing to assist in the recruitment process they were supplied with written information about the study and leaflets for parents printed in German or Turkish. To recruit Turkish participants a local group of Turkish women was contacted. Mothers or fathers willing to participate were called by a member of the research team to inform them about the study procedure, their rights related to the participation and to assess their eligibility.

### Data collection

Focus groups were held in two Basel area community centers, one urban location and one suburban location. The focus group discussions were held between June and November 2011 and lasted 180 min, on average and babysitting was provided. A nursing or midwifery researcher moderated the discussion, and an assistant (midwife or health visitor) assisted with the documentation – yet professional roles were not revealed to participants to avoid a potential social desirability bias. German moderators were trained conducting focus group discussions combined with the ‘playful design’ method. The first authors trained the Turkish moderator and assistant and supervised the conduction of the Turkish focus group. The discussion guide was pretested in a pilot group and was deemed acceptable without further necessary modification.

In brief, the playful design comprised inviting participants to use three-dimensional objects (e.g. plastic bricks, figurines) to present individually, then collectively their perspective on the support during the transition home following childbirth.

We incorporated a ‘warm-up’ introduction to the playful design method wherein participants were invited to create ‘summer holidays’ with the bricks and figurines to understand how to create metaphorical attributions (e.g. yellow brick represents the sun). Subsequently, participants constructed a representation of their individual day-to-day life as mother/father and of their experiences with postnatal care. After discussing these individual views in the group, they created a group response to the following question: “Imagine you have been discharged on the third day after the birth of your child. Using the plastic bricks and figurines, please construct a model of postnatal care that best meets your individual needs after hospital discharge.” Participants used their individual models (and attributed metaphors) to construct a group model that was discussed and negotiated between all participants. This constructive approach, allowed us to examine the physical representation and ‘sense making’ of individuals and the group. Group discussions were audiotaped while the assistant recorded field notes (i.e. group interactions, nonverbal communication) and took photographs of the constructed models.

### Data analysis

**Fig. 1:**
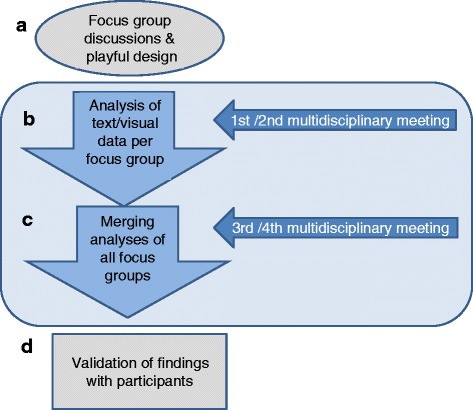
Diagrammatic plan of analysis: **a** Focus group discussions were recorded and photos taken of the playful design elements. **b** All text and visual data were coded by two investigators in an iterative process, steadily comparing emerging codes until consensus of a final code book was reached. Codes were then sorted into themes. During this process, two multidisciplinary meetings were held for in-depth discussion of the data, refining the code list, and reaching consensus on central themes. **c** The two investigators compared the findings from all focus groups to identify recurrent patterns. During two multidisciplinary meetings the team members deepened the comparative analyses and developed a thematic map of the central topics and the relationships between central themes. **d** The integrated findings were vetted with the representatives of the focus groups participants

Audio recordings were transcribed verbatim and potentially identifying data were removed (and pseudonyms were assigned) to protect participant confidentiality. The verbatim transcription of the discussions was validated by the research team, along with the field notes and photographs. An adapted form of thematic analysis, as described by Braun and Clark [30], was used to analyze the transcripts from the focus group discussions and pictures of the constructed models. Briefly, thematic analysis aims to systematically code the data, identify themes, analyze them and search for recurrent patterns (Fig. 1).

The software Atlas.ti was used to code the data and to sort the codes into themes. In an effort to avoid missing important themes, all data were coded by two investigators. For the report of the findings, all selected quotes were translated by one author from German to English. The translation was validated by a bilingual speaker with research experience.

For the analysis of the Turkish focus group data, we organized an extra session with the Turkish moderator (midwife) and the Turkish-German interpreter who assisted with the conduct of the focus group and who translated the group discussion.

### Ethics considerations

The study was reviewed and approved by the local institutional review board (ethics Committee) in Ethikkommission beider Basel, Switzerland (Ref. No. EK 99/11) and all participants provided written informed consent prior to participation. The participants were informed about their right to withdraw and stop their participation without needing to explain the reason. All participants were provided with a list of relevant resources and services should the focus group discussion trigger adverse reactions in relation to the birth and the postpartum period.

## Results

We conducted six focus group discussions. Four discussion groups were held with German speaking mothers (*n* = 18), one group with Turkish speaking mothers (*n* = 2) and one group of fathers (*n* = 4). Nearly all participants experienced the birth of their youngest child between 4 weeks and 9 months prior to data collection (one mother had given birth 16 months prior). The socio-demographic and obstetric characteristics are summarized in Table 1.

**Table 1:** Demographic characteristics of the participants (*n* = 24)

Characteristics	No. (%)
Age	*n* = 23^a^
	mean 32.7 ± X yrs
	range 26–43 yrs
Sex	*n* = 24
Female	20 (83 %)
Male	04 (17 %)
Professional education	*n* = 24
> 4 yrs	14 (58 %)
2–4 yrs	09 (38 %)
< 2 yrs	01 (04 %)
Origin	*n* = 24
Swiss background	16 (67 %)
Migrant background^b^	08 (33 %)
Family situation	*n* = 24
Partnered	23 (96 %)
Single	01 (04 %)
Parity	*n* = 24
One child	21 (88 %)
Two children	02 (08 %)
Three children	01 (04 %)
Place of delivery	*n* = 24
Hospital	22 (92 %)
Birthing center	02 (08 %)
Mode of delivery	*n* = 24
Vaginal	06 (25 %)
Assisted vaginal	08 (33 %)
Cesarean	10 (42 %)
Duration of hospital stay	*n* = 23^a^
> 5 days	06 (26 %)
4–5 days	14 (61 %)
≤ 3 days	03 (13 %)
Midwifery care at home	*n* = 24
	19 (80 %)
Use of community-based health visitors	*n* = 23^a^
	20 (87 %)

^a^unknown for one participant ^b^Germany, Turkey, Iceland, South Africa and two double nationalities not counted in the statistic (Switzerland-France, Switzerland-Netherlands)

**Fig. 2:**
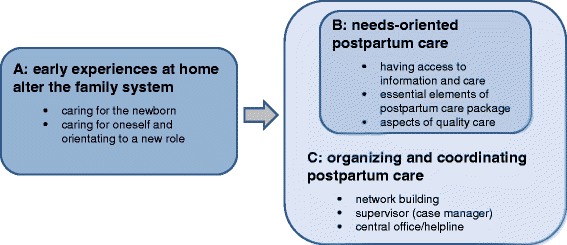
Thematic map of main themes and subthemes

Analysis of the focus group discussions revealed themes relating to three main topics: (A) depicts the situation of new parents and their newborn after the transition to home; (B) reflects the parents’ views on dimensions of care meeting their needs; and (C) the organization of postnatal services that would match their needs (see Thematic map, Fig. 2).

### A: early experiences at home alter the family system

The birth of a child led to major changes within the family system. First-time parents frequently reported feeling overwhelmed by their new responsibilities:

*“It’s like an astronaut in a room void of air. You can prepare and attend a course. But then the child is born, bang, and everything is new. Nothing is in the right place anymore. And we are standing somewhere and are trying to get our feet back on the ground. “(Esther, 31 years, first child, 2.5 months, caesarian delivery).*

These parents stated that they felt a huge gap between what they had imagined and the reality of the postpartum period. Central emergent subthemes from the discussions related to caring for the newborn, caring for oneself and orienting oneself to the new situation.

#### Caring for the newborn

Newborns demand parental attention for feeding and care. For most parents, feeding was considered a complex task; this led to feelings of uncertainty and desire for additional guidance. Initiating breastfeeding was particularly challenging and new mothers cited insecurity about providing a sufficient number/amount of feedings as well as worries about using the correct technique. When confronted with the hypothetical scenario of an early hospital discharge, many mothers expressed fears about their ability to breastfeed: *“I would not have been able to breastfeed after 2 days. I think I would have had to give the bottle. He was crying so much in the first days and I didn’t know what to do because there was no milk yet.” (Christiane, 32 years, first child, 7.5 months post-instrumental delivery).*

Parents reported feeling insecure and sometimes unprepared to care for their newborn. These feelings would be magnified by early hospital discharge. *“In the hospital one learns quite a few things about baby care, how to wash, dress and feed the baby. Can this still be guaranteed after an early discharge?” (Anna, 35 years, first child, 3 months, vaginal birth).*

Parents felt the weight of their new responsibility, particularly in situations when they were expected to assess the baby’s health status. *“We were worried in many situations. Our daughter often woke up startled and pulled her head backwards. We didn’t know what this meant and as she was stuck in the birth canal, we were afraid of a birth trauma.” (Felix, 36 years, first child, 4 months, cesarean delivery).*

#### Caring for oneself and orientating to a new role

Parents reported that the newborn determined the daily routine and parents described how meeting their own needs became secondary: *“I had to meet the baby’s needs and go with its rhythm and I was only number two, the whole time “(Barbara, 36 years, second child, 8 months post-vaginal birth).* This was often evident regarding their diet as many had difficulty in eating regularly: *“Even though our baby was healthy and all went well, we repeatedly realized late in the evening that we hadn’t eaten dinner yet.” (Fabian, 33 years, first child, 4 months post- instrumental delivery).* Not eating regularly (or not eating a healthy diet) was often accompanied by feelings of guilt related to the potential negative effect on breast milk quantity/quality: *“In the end I realized, hey, I need to eat and produce milk.” (Barbara, 36 years, second child, 8 months, vaginal birth).*

Because of perineal birth trauma or caesarean delivery, women experienced pain and constraints to their physical mobility. This limited them taking on all the tasks expected of a new mother. Initially, some were unable to carry their baby or put them on the breast without assistance: *“In the first 2 weeks I was not able to hold my child without pain. And walking was impossible; I was curved as a banana.” (Aline, 29 years, first child, 1 month, caesarean delivery).* Others found routine housekeeping, shopping or medical appointments to be very challenging *“Four weeks after the delivery I had the first appointment at the pediatrician’s and I remember that this seemed to be huge to me. Oh, Monday is approaching, how on earth will I be able to manage this? Yes, something you’ve done very easily before suddenly is a big challenge.” (Amina, 31 years, first child, 6 months, instrumental delivery).*

Sleep deprivation in the first weeks and months was frequently cited as problematic: *“This was probably the most extreme, not to sleep. But if you sleep so little, you eventually don’t even feel hungry anymore.” (Esther, 31 years, first child, 2.5 months, caesarean delivery).* Feeling fatigued and exhausted, and the resulting impact on mental wellbeing, were particularly evident in cases of colic or excessive infant crying. These mothers reported heightened psychological burden and noted symptoms of postpartum depression. *“I would have been too proud to admit that I have a problem, that I am suffering an awful lot due to sleep deprivation and not being able to stop the baby from crying. I remember how dreadful I felt [after the birth of the first child] when I had to write on the birth announcement cards how happy we are to be the three of us now and that was not true.” (Barbara, 36 years, second child, 8 months, vaginal birth).* Some mothers also remarked that the attention showed on the child left them feeling frustrated that their own needs were secondary: “*How can a mother ‘mother’ her child if nobody mothers her? It starts with the mother and then she can pass the energy to the baby.” (Aline, 29 years, first child, 1 month, caesarean delivery).*

Women reported relying heavily on their partners/family in the first few turbulent weeks at home. Most fathers expressed a strong desire to support their partners and be in contact with the child: *“My wife had a difficult delivery and needed a lot of support. As she was unable to get up, I had to bring her the baby every 2 h for breastfeeding. And I changed the nappies, did the laundry, cooked and very seldom, when I found time, I did the shopping.” (Felix, 36 years, first child, 4 months, caesarean delivery).* However, not all participants could draw on this source of support to the same extent and needed time to adapt to their new roles. *“When my husband had to go back to work it just became too much for me. I had the chance of staying with my parents-in-law for 2 weeks. This allowed me to spend my time with the baby and to establish a better connection between us. I got into something like a rhythm and then the whole thing became a bit more structured.” (Esther, 31 years, first child, 4 months, caesarean delivery).* Parents expressed feeling pressure to meet what they felt to be an unrealistic, idealized role as a new mother: *“Sometimes one feels a real pressure from society to be able to manage everything alone.” (Anna, 35 years, first child, 3 months, vaginal birth).* Indeed, some expressed a reluctance to ask for help for fear of failing in their new role: *“I felt inhibited to ask for help, because I thought that I had to manage it on my own”. (Amina, 32 years, first child, 6 months, instrumental delivery).*

Despite challenges and frustrations, parents also acknowledged positive aspects: “*It can be very nice going home after two days and spending the whole time with the child in a well-known and intimate place.*” *(Emma, 28 years, first child, 4.5 months, vaginal birth).*

### B: needs-oriented postpartum care

Parents expressed the need to have a “safe start” as a family at home. Participants agreed that postpartum care should be easy to access. Moreover, it should offer a well-defined package of services that contribute to building security, allowing parents to get rest and to gain confidence in their new situation: *“We should not have to beg for everything, but rather know that this is what I have behind me [support] and if I need it, it’s there. That, as a mother, I’m not expected to care for everything myself and that I’m allowed to lie down at some point.” (Anouk, 37 years, first child, 8.5 months, instrumental delivery).*

#### Having access to information and care

Parents expressed their desire to be informed early about available postpartum care and how to access it: *“A guideline should be available at the beginning of the process [pregnancy] and give information on what to expect when one becomes a parent.” (Felix, 36 years, first child, 4 months, cesarean delivery).* Birth preparation classes or direct contact with health professionals were the preferred information sources: *“You can read a lot or get information through other sources, but it’s quite different if you can hear and learn from a health professional [in a birth preparation class]. If there are too many sources, there’s a risk of getting incorrect information.” (Dilek, 26 years, first child, 16 months caesarean delivery).* The mothers frequently accessed the internet for information, yet parents found it difficult to judge the quality of the information and stated that they would prefer a site moderated/reviewed by health professionals. The groups also suggested a telephone hotline as a convenient source of information: *“I would very much appreciate, if there were a contact point, such as a telephone hotline, where I could just call and get the information.” (Constance, 43 years, first child, 2.5 months, caesarean delivery).*

Some participants expressed difficulty in accessing care reporting that it was difficult and time consuming to organize postpartum care: *“I spent an eternity on the phone to find a midwife. I called all of the midwives on the city list, but I couldn’t reach a single one of them.” (Anna, 35 years, first child, 3 months, vaginal birth).* For some parents, they expressed their wish for an optional postpartum care package offered to all families after hospital discharge: *“It would be best for the mother if the offer would come automatically and she could say yes or no to it.” (Amina, 31 years, first child, 6 months, instrumental delivery).*

In addition to the organizational aspects of accessing care, participants also noted restricted mobility with the new baby at home. These, mothers expressed a strong need for home visits that would contribute to the family’s sense of wellbeing and security: *“If we are sent home so early, then the health professionals should do home visits. This is important for our security.” (Dudu, 26 years, first child, 3 months, caesarean delivery). “Already, the thought that the health professional will come today or tomorrow would encourage us.” (Dilek, 26 years, first child, 16 months, caesarean delivery).* Home visits of sufficient frequency and duration were seen as essential in the first 2 to 4 weeks post-delivery. *“The midwife’s home visits are very important for the mother and her child. She should come longer than 10 days, longer than an hour per visit and if needed several times a day.” (Anouk, 37 years, first child, 8.5 months, instrumental delivery)* (Additional file 1: Picture 1).

Participants also described emergencies requiring immediate professional attention and 24-hour access to care. *“My nipple was bleeding and she somehow drank the blood and it then ran out of her mouth. I was totally in a panic and I would have absolutely needed to talk to someone.” (Anna, 35 years, first child, 3 months, vaginal delivery).* Overall, the threshold for calling a health professional seemed high, either because the situation occurred outside of consultation hours or because parents were unsure of whom to contact. A core request across all groups was access to a 24-hour helpline for urgent situations. *“We needed to have a health professional we could call in case of an emergency.” (Anouk, 37 years, first child, 8.5 months, instrumental delivery).*

#### Essential elements of the postpartum care package

The care needs expressed in the focus groups fell into 3 broad categories: monitoring the health and wellbeing of mother and child, counseling services, and domestic assistance.

Parents desired a follow-up health professional home visit to assess and monitor the health status of both mother and child: *“I needed the midwife to check my suture and to keep an eye on the baby in order to assure that everything was alright” (Aline, 29 years, first child, 1 month, caesarean delivery)."* (Additional file 2: Picture 2) for some mothers, it was very important that the health professional screen for deviations from the norm, such as exhaustion or onset of postpartum depression: *“I was too proud to admit that I had a problem. But if a health professional would have called or asked the delicate questions, it would have bubbled out of me. I would have needed help very urgently.” (Barbara, 36 years, second child, 8 months, vaginal birth).* Parents also expected health professionals to take action, to follow up and make referrals as needed: *“When I had an inflamed breast, the midwife gave me advice and came back later to check whether it was better.” (Anouk, 37 years, first child, 8.5 months, instrumental delivery).*

Participants wanted more tailored health information, consultation and guidance in their new roles as parents: *“It was so important that somebody came to my home and saw our situation. Somebody who observes breastfeeding and gives tips: If you are breastfeeding like this, it might be useful if you put your legs on a stool like this. There had been so many things I did not pay attention to and that nobody told me in the hospital. They only became an issue when my breast started hurting.” (Anouk, 37 years, first child, 8.5 months, instrumental delivery).* Parents also wanted to be empowered to make the right decisions regarding their health, their child’s and to be able to recognize early signs of health problems: *“Information is very important. I had inflammation of the breast. I did not even know that this existed. Or when the lochia [postpartum bleeding/secretion] is not good anymore. Nobody told me that the color would change. This is information I expect to be given in order to be able to recognize the signs [of a problem].” (Emma, 28 years, first child, 4.5 months vaginal birth).*

Besides individual counseling, it is important for health professionals to observe parents as they started to take on new tasks, and to offer affirmation and help them make sense of their new parenting experiences: *“I was always so grateful when she was ringing the bell. Even if she was just observing and then telling me how good my child and I were doing. Or giving me some advice or helping me understand my child who did cry a lot. This was so important to me and I still benefit from this now." (Amina, 32 years, first child, 6 months, instrumental delivery).*

As part of the postpartum care package, the possibility of domestic assistance was highly valued - especially for single mothers without sufficient family support. During the first days at home, one single mother recovering from a caesarean delivery was barely able to bend to pick up her baby. Further, partnered parents also wished more support. *“A domestic aid would be good. Someone who does the shopping, cooks, and cleans the house. During the first 2 to 3 weeks, I had difficulties with my circulation and my episiotomy was so painful I could hardly move.” (Emma, 28 years, first child, 4.5 months, vaginal birth).* A number of new parents expressed sentiments that household assistance such as regular provision of healthy food might have a positive impact on the mother’s recovery and on their ability to cope with the new situation.

#### Aspects of quality care

For new parents, continuity was a major factor in determining quality of care. This includes having a health professional who knows the family and the health history, who establishes a bond of trust, offering care oriented to the family’s specific needs: *“The midwife caring for me at home already followed me through my pregnancy, she somehow managed the case. She was my contact person and I felt extremely well looked after anytime. Because she knew the whole history, it all felt very personal.” (Bernadette, 32 years, first child, 2.5 months, instrumental delivery).* Some parents saw a potential benefit in an early discharge with guaranteed postpartum care at home. They felt that this might help facilitate an improved continuity of care compared to the regular shift changes experienced in the inpatient hospital care setting.

New parents shared impressions that current postpartum care services are comprehensive, but fragmented: *“Currently, I perceive it as quite scattered. The midwife has nothing to do with the pediatrician and nothing to do with the parent counselor/mother and father counselor. All over the place are isolated persons with their competences and in between is almost nothing.” (Amina, 32 years, first child, 6 months, instrumental delivery).* For these parents more integrated information and care was important to minimize feelings of disorientation and confusion: *“The different health professionals sometimes had conflicting views. And if everything is new to you, this is quite confusing. I would appreciate if it would be united a bit." (Anouk, 37 years, first child, 8.5 months, instrumental delivery).* It was also critical that participants’ health history was well documented and effectively circulated within the system to ensure coherent care.

Although parents wanted a standardized postpartum care package offered to all families, they also desired a family-centered, needs-orientated approach. This was particularly relevant concerning breastfeeding as many mothers expressed negative experiences with a uniform approach to care, preferring more flexible guidance: *“If breastfeeding is not successful, it seems important to me that a heath professional does not take a rigid attitude. She should be open and flexible and see the individual need of the woman. If one way doesn’t work, there is another way which is also good for the child.” (Constance, 43 years, first child, 2.5 months, caesarean delivery).*

### C: organizing and coordinating postpartum care

Participants in each group were asked to collectively design a model of postpartum care that would meet their needs. Despite variations across groups, three main organizational strategies could be identified: network building, supervisor/case manager and a coordinated helpline.

#### Network building

**Fig. 3:**
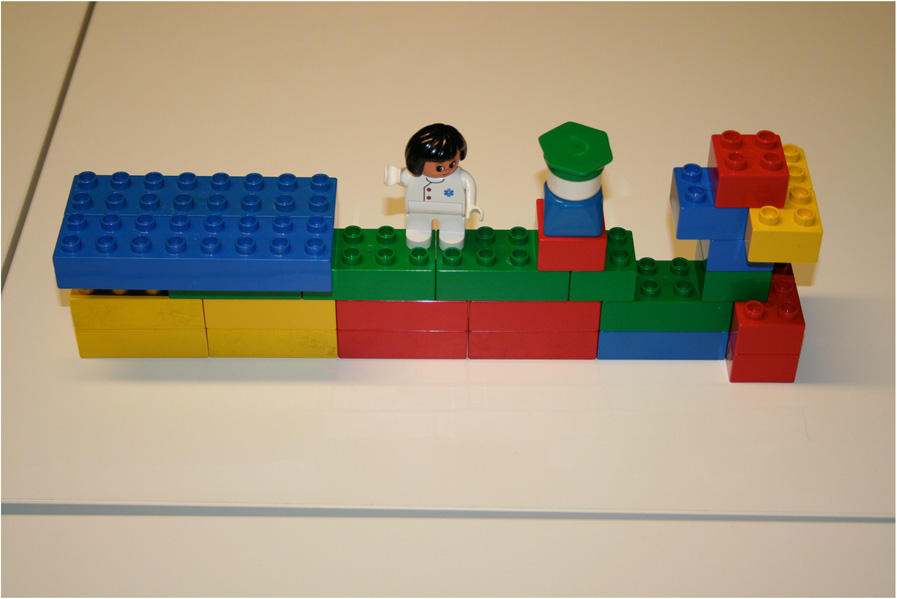
Care model developed by fathers: Process-orientated illustration of pregnancy (green), delivery (red) and postpartum (yellow) where the points of intersection between health professionals overlap, preventing parents from falling through the network

Parents would like to see health professionals better connected to each other and working collaboratively. The fathers expressed wanting to clearly understand each provider’s respective competencies and see coordinated care: *“The bricks, this is the network and you can see that they are interconnected. The transitions are overlapping, it’s working.” (Felix, 36 years, first child, four months, caesarean delivery)* (see Fig. 3).

**Fig. 4:**
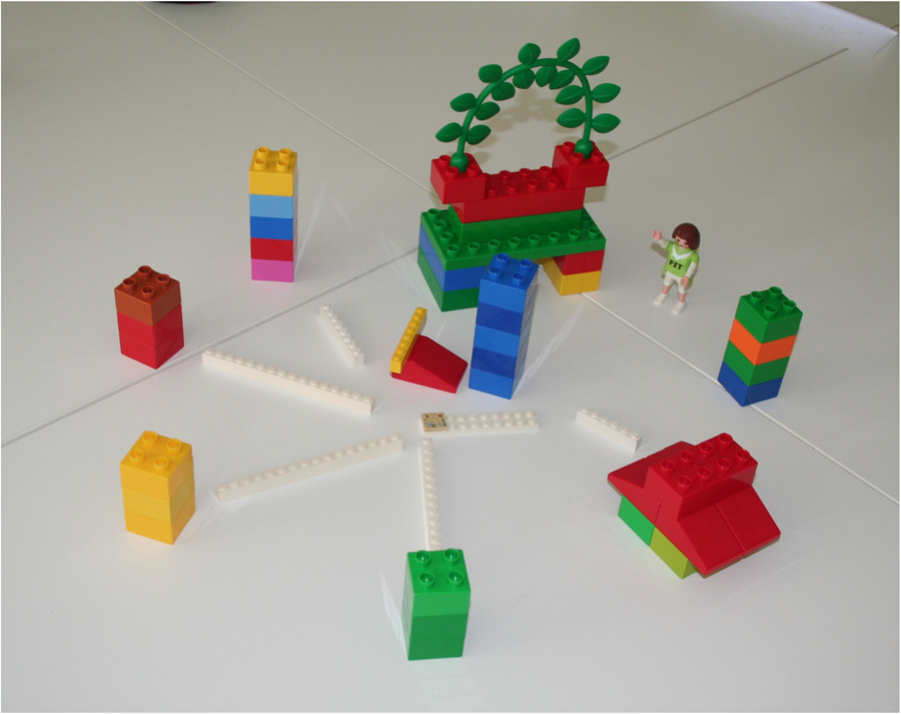
Care model developed by German-speaking, first-time mothers: The health professionals surround the mother (“sitting on the throne” – red seat with plants) and are connected with white strips, creating a network with clear lines of communication

German speaking, first-time mothers built a network represented by white connecting strips, ensuring that the various health professionals are connected to each other with clear lines of communication, permitting effective data flow (see Fig. 4).

#### Supervisor (case manager)

Most collaboratively created models included a type of case manager role: *“That we have somebody who has it in her hands a bit and who is somehow responsible for us. Someone who keeps the overview, a supervisor… She does not need to be able to do everything on her own, but she needs to know who to mobilize if needed. Somebody who realizes: Attention, this small flame could flare up." (Barbara, 36 years, second child, 8 months, vaginal birth).*

Mothers felt that the supervisor should be a health professional with a broad range of postpartum care skills and have the ability to recognize when to mobilize additional support and know where to find it: *“It would be good if one already knew in pregnancy who the midwife will be, who’s coming for postpartum visits. The midwife would have to come to our home in the first 4 weeks following the delivery and we would have to be able to reach her by phone, also during the night. In addition, she should have the knowledge to decide whether she can answer a question or whether it is a situation, she needs to refer to a gynecologist or pediatrician." (Anouk, 37 years, first child, 8.5 months, instrumental delivery).* The metaphor of the bridge was used in two models to illustrate the role of a case manager in connecting the mother and her child with other health professionals and health institutions (Additional file 3: Picture 3).

#### Central office/helpline

A recurrent feature of the models was a central office or helpline, easily accessible 24-hours a day: *“In the beginning it is good if one has somebody 24-hours a day, something like a telephone hotline, a central office." (Esther, 31 years, first child, 2.5 months, caesarean delivery).* Others mentioned a role for a centralized triage office for communication and sharing information: *“There should be a midwife-operated hotline, which would come out of this midwifery knowledge pool. Moreover, as they know their limits, they would know when a doctor would know better. It would be good not to have too many contact points. It should be bundled a bit.” (Amina, 32 years, first child, 6 months, instrumental delivery).* Furthermore, the central office would facilitate and organize postpartum care. *“In the beginning, making a phone call was difficult for me as the situation simply was too turbulent. I delayed it and never did some things. Besides, I was often unable to reach somebody and would have had to call somewhere else again. Therefore, I would find it helpful if the central office would facilitate the organization of care." (Constanze, 43 years, first child, 2.5 month, caesarean delivery) *(Additional file 4: Picture 4).

## Discussion

This study utilized focus group methodology with a playful design element to explore new parents’ experiences during the initial postpartum transition home. This approach elicited new parents’ conceptual views on care models that would best meet their needs following early hospital discharge. Parents reported feeling overwhelmed at times by the responsibilities as the primary caregiver for the newborn and having to attend to her own self-care needs while recuperating from birth. This resulted in significant practical and medical support needs. Participants were critical of the access to postnatal care services and the lack of coordination. They desired more coordinated postnatal care that was readily accessible including home visits, a 24-hour helpline, and potentially, domestic aid. These findings may reflect the problem of fragmented services in modern health care systems, and point to the care and support needs of new parents and newborns during the sensitive period following early hospital discharge. Notably, these needs may be magnified by changing family structures in society as well as cross-border migration resulting in socially more isolated families lacking support from extended family. Many health care systems will not have the resources to meet all these needs, the focus group discussion findings provide insight into needs and spur reconsideration and a prioritization of health resources across the spectrum of postnatal health services.

### Early experiences at home alter the family system

The challenges described by participants in taking on full responsibility for a newborn is consistent with findings from previous studies [31]. First-time parents often experience feelings of insecurity, fear and self-doubt associated related to their lack of parental experience [32–34]. There is extensive evidence that the round-the-clock care required by newborns limits parental rest as 46–87 % of new mothers reporting feeling fatigued which can result in developing symptoms of depression [35, 36].

To our knowledge, the finding that newborn parenting may hamper parents’ food-intake has not been described so far. Yet given the demands of infant care and the self-care requirements on new mothers, this is perhaps not surprising. In the nuclear family, the new father is an important source of support for mother and child [37]. Although fathers participating in parenting studies seem highly motivated to take on an active role, it is challenging for them, especially when they have to balance family and job demands [31, 38].

In many societies, extended family members [39, 40] provide postnatal support. Participants in our study expressed that our society expects new parents to manage life with a newborn without additional help, posing a barrier to receive social support. Indeed, there is a growing body of literature on the negative impact of diminished social support on new mothers’ mental health [41, 42]. The present findings suggest that one must not only assess social support but that perceived social norms that constrain new parents’ help-seeking behavior are also worthy of consideration.

Physical recovery following vaginal birth or cesarean section was described as brought additional burdens such as pain and reduced mobility. Previous studies have demonstrated this hampers child care and activities of daily life – particularly following cesarean delivery [43, 44]. Participants in our study complained of constrained mobility after vaginal birth as well. Although prolonged bedrest after birth is no longer recommended, new mothers’ need for recuperation and rest may need to be reconsidered [45].

### Needs-oriented content and quality of postpartum care

New mothers and fathers in our study emphasized the importance of having a competent person at hand for monitoring maternal and child health as well as for counseling/support. They considered home visits by a midwife and a 24-hour helpline to be important elements of postnatal care. The need for guidance from an experienced person in the early days of caring for a newborn has also been found in several previous studies [46, 47]. The availability of professional support has been shown to be important for reinforcing parental comfort level and feelings of security [20, 23, 48, 49], minimizing parents’ experiences of strain [31] and individually-tailored home visits help reduce postpartum depression rates [10, 50]. In each focus group, participants emphasized that the professional home visits alleviated their concerns, conveyed parenting skills and enhanced their parental self-confidence, confirming findings from previous research [9, 49, 51]. Indeed, recent recommendations from the WHO recommend postnatal home visits as an evidence-based measure to improve maternal- and child health [52]. Importantly, professional support was not seen as beneficial when the counseling style was directive or conflicted with advice from other professional caregivers, findings mirrored in previous studies [23, 53, 54].

The desire for domestic assistance was strongly expressed during group discussions, confirming findings of another Swiss study [53]. The basic health insurance plans ceased reimbursement of home domestic aid in 1996, and since then most Swiss childbearing families do not have this assistance during the postnatal period [33]. In the Netherlands, domestic support is still widely used [55]. Similar support systems have been studied in the UK [56] and in Australia [57] and show high user satisfaction, yet positive effects on maternal health have not been clearly demonstrated. The desire for professional domestic aid may also be related to the fact that traditional postnatal support from the extended family has nearly disappeared in postmodern societies where – due to societal change and migration - most new parents live in small nuclear families [58]. New social norms stress the importance of independency [59] and model the ideal mother as self-reliant and selfless. Such normative beliefs may hinder new mothers to ask for and to accept help within their social network [60, 61].

### Accessible, coordinated postpartum care

Although the mandatory health insurance in Switzerland guarantees universal access to health care, participants described problems identifying and accessing outpatient services after hospital discharge. Systematic collaboration between hospitals and community-based services is lacking in the Swiss health care system in general [62]. Continuity of care is further hampered by the lack of interconnectivity between different outpatient health care services and inability to readily share patient health information (i.e. interconnected electronic health records) [63]. A prior evaluation of Swiss postnatal care identified fragmentation and lack of continuity of care as major problems and noted that gaps often result from a shortage of community-based midwives [24]. Our focus group discussions echoed these points. Poor coordination and suboptimal follow-up care for new mothers and infants have been observed in other high-resource countries including the U.K. [54], Sweden [23, 64], Canada [65] and Australia [66]. Notably, a Canadian study across four regions revealed that areas with greater inter-organizational collaboration had the most accessible and better continuity of perinatal health services [67].

### User-suggested coordination of postnatal care

Participants in our study identified three potential strategies for enhancing coordination of postnatal care: network building, installing supervisors, or establishing a central office/hotline.

One group of mothers suggested a *network model* similar to the one described by Willumsen and colleagues [68]. This approach involves network building that leverages existing organizational units and adapting communication processes to transfer information between different health services. The model proposed by the fathers’ group in our study went one step further by building a continuous care pathway from pregnancy to the postnatal period, interlinking the different care providers in a systematic manner. This would represent an integrated care pathway, which ‘gains the perspective of the service user journey’ [69]. Barimani and Hylander reported efforts to improve continuity in maternity care by connecting midwives and child health nurses in the Swedish healthcare system using a common ‘chain of care’ resulting in smoother transition between services with resulting patient benefits [64].

Some focus group members suggested the use of a supervisor or case manager. This approach is rarely employed as the demands of the role are seen as too comprehensive given the volume of patients. In the U.S., obstetrical nurse case managers have been used to manage high-risk patients [70] or vulnerable, disadvantaged families [71]. In many countries, midwives have case management responsibilities in perinatal care – a role noted by several participants in the present study. A recently updated Cochrane review showed that midwife-led continuity models of care for childbearing women had positive effects on health outcomes, cost-reduction and client satisfaction [72].

There is limited evidence on the use of *telephone hotlines* in the postpartum period. One American study found that only 28 % of new parents utilized a helpline staffed by the postpartum ward [73] and a similar percentage (24 %) used a midwife-run helpline in Lebanon [74]. This intervention, yielded lower stress scores among first-time mothers [75]. Frequent concerns relate to breastfeeding, infant care and nutrition [74, 76]. Most calls could be managed by nurses with fewer than 1/5 (18.6 %) of calls requiring referral to a physician [74]. One Canadian study described a helpline offered by public health nurses that not only answered parental questions but also provided information and linked parents and professionals alike to community resources [76]. This model seems to be closest to what the participants in our study envisioned — a ‘central office’ capable of triage and referring parents to different services.

Notably, no participants in our study mentioned postnatal telephone support programs [77] or video conferencing [78] – likely, because such services are uncommon in the Swiss perinatal health services.

### Limitations and strengths of the study

Study findings should be interpreted with caution given the limited number of participants. A major challenge in this study was recruiting the intended sample representing a broad range of postnatal experiences. Given the political challenges of migrating populations in Europe, information on migrant patients is important. The Turkish mothers were difficult to reach given language barriers and no Turkish speaking fathers were included. Indeed, the smallest groups were the fathers (*n* = 4) and Turkish speaking mothers (*n* = 2) thus limiting the transferability of these specific results. While these perspectives are important, we are reluctant to make any special recommendations given this limited sample. Another limitation related to the sample is the high proportion of first-time parents who had vaginal instrumental or cesarean births; a population that may express more post-discharge care needs than experienced parents or mothers who have vaginal births. Nevertheless, recruitment through various professional groups guaranteed that participants reflected a broad range of experiences with existing postnatal care services.

During data collection, a possible limitation was related to the fact that focus groups were mainly conducted by midwives, sometimes supported by a nurse or health visitor. To minimize this bias, and avoid an overemphasis on midwifery, moderators introduced themselves as researchers rather than midwives. Additionally, data were analyzed by a multi-professional group, consisting of practitioners and researchers from nursing, midwifery and medicine to allow a more diverse interpretation and thus may be a considered as a relative strength of the study.

The combined focus group and playful design allowed us to both document the progression of the group discussion on models of care and promoted consensus building. However, not all participants were equally confident in suggesting steps for refining the care model, and in some groups, a single person took on a leading role. This underscores the importance of appropriately training the moderator to manage such group dynamics. Nevertheless, we consider the integration of interview text and pictures of the constructed models that allowed for reciprocal validation as an appropriate methodological approach to minimize this potential limitation. The major strength of this study was to offer new parents the opportunity to freely envision complex models of care that would meet their needs using a medium that provided a means of expression for those who may not feel comfortable speaking.

### Implications for practice and further research

To optimize follow-up care after early postnatal hospital discharge it appears that health systems must look beyond the traditional silo approach. Societal changes continue to reshape families and migration throughout Europe is transforming traditional family structures. Effective transition from hospital to home requires that care providers develop organizational infrastructures for collaboration and effective data sharing and communication. Families may benefit from a more integrated postnatal care package that includes home visits from qualified health professionals, access to a 24-hour helpline, and, in the absence of social support - affordable domestic assistance (if needed). Such a safety net could help support the recovery of the motherpost-childbirth, promote self-care and empower new parents in their new role as primary infant caregiver. Future research is needed to elucidate the institutional barriers limiting the interface between hospital and different community-based services, while taking into account new parents’ experiences and needs on their journey through the maternity care system. In addition, future studies could investigate specific needs of inexperienced parents, mothers after operative deliveries and immigrant families as these may be the most vulnerable populations.

## Conclusions

Returning home from hospital with a newborn child is a challenge that results in significant needs for practical support and health monitoring for mother and child. Even in a high resource country like Switzerland, access to follow-up care is not guaranteed due to the lack of coordination of services. To meet their needs, new parents desire integrated care services, a 24-hour helpline, home visits by qualified health professionals and the possibility of domestic support.

## Additional files

Additional file 1: Picture S1. Care model developed by Turkish mothers: Midwife stands at the mother’s bed and makes regular home visits. Other health professionals (in the yellow car), such as a pediatrician, gynecologist and psychologist are on call and offer home visits if needed. (JPG 1231 kb) Additional file 2: Picture S2. Illustration of the health professional keeping a watchful eye upon the health and wellbeing of mother and child. (constructed by Aline, 29 years, first child, 1 month, cesarean). (JPG 426 kb) Additional file 3: Picture S3. Care model developed by German-speaking, first-time and multiparous women: Mother, child, father and midwife are in the center. The red bridge illustrates the role of the case manager linking the family to the other players. (JPG 1484 kb) Additional file 4: Picture S4. Care model developed by German-speaking, first-time mothers: The family and the health professionals are arranged around the central office. The watch represents 24-hour availability and the computer in the foreground represents the telephone and bundled information available from the central office. (JPG 2982 kb)
