# Supplementary figures and images for: Safe start at home: what parents of newborns need after early discharge from hospital – a focus group study
Source: BMC Health Serv Res. 2016 Mar 8;16:82. doi: 10.1186/s12913-016-1300-2 (PMC4782306; doi:10.1186/s12913-016-1300-2)

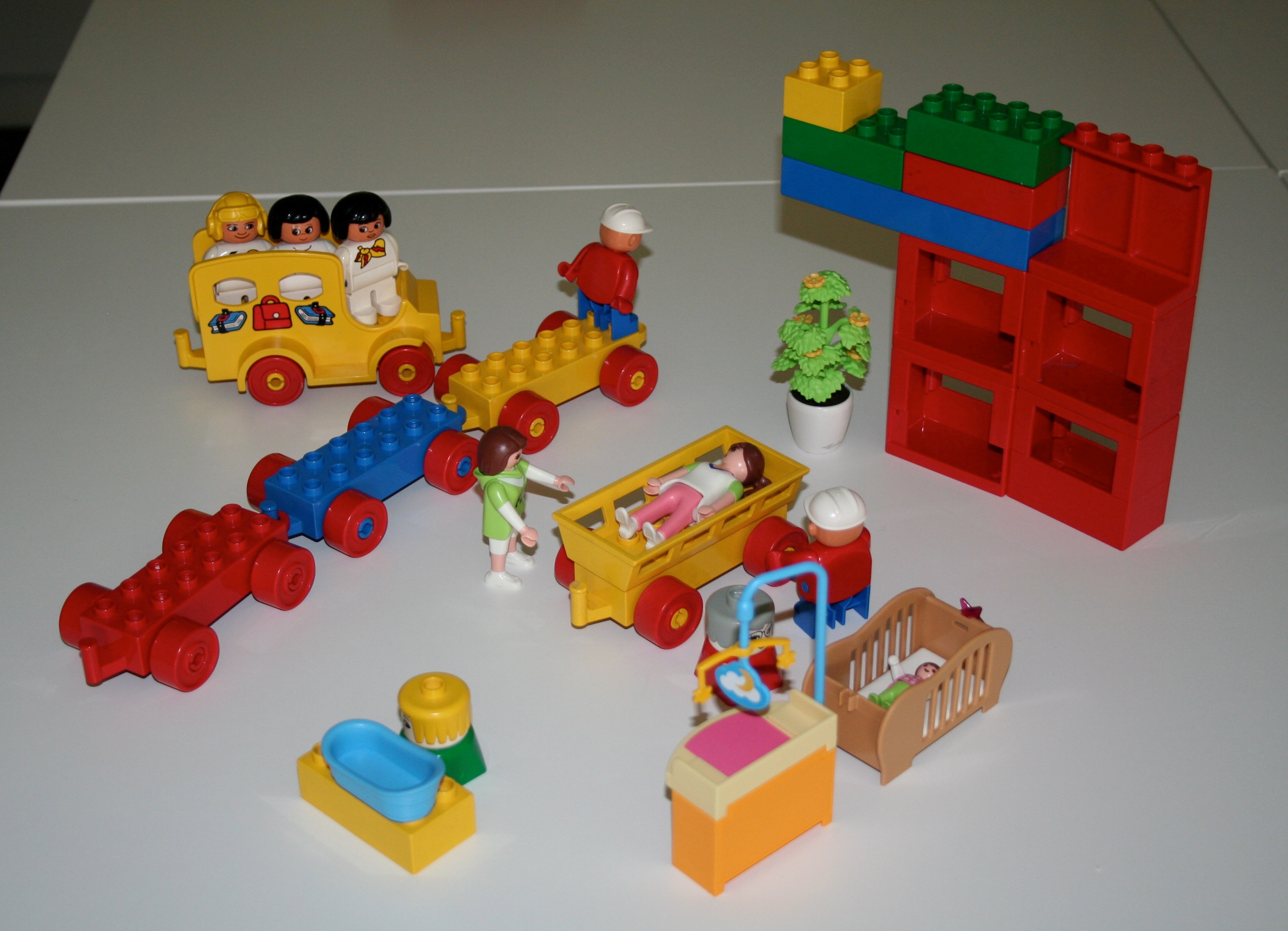

Supplement: Additional file 1: Picture S1. — Care model developed by Turkish mothers: Midwife stands at the mother’s bed and makes regular home visits. Other health professionals (in the yellow car), such as a pediatrician, gynecologist and psychologist are on call and offer home visits if needed. (JPG 1231 kb) [file 12913_2016_1300_MOESM1_ESM.jpg]

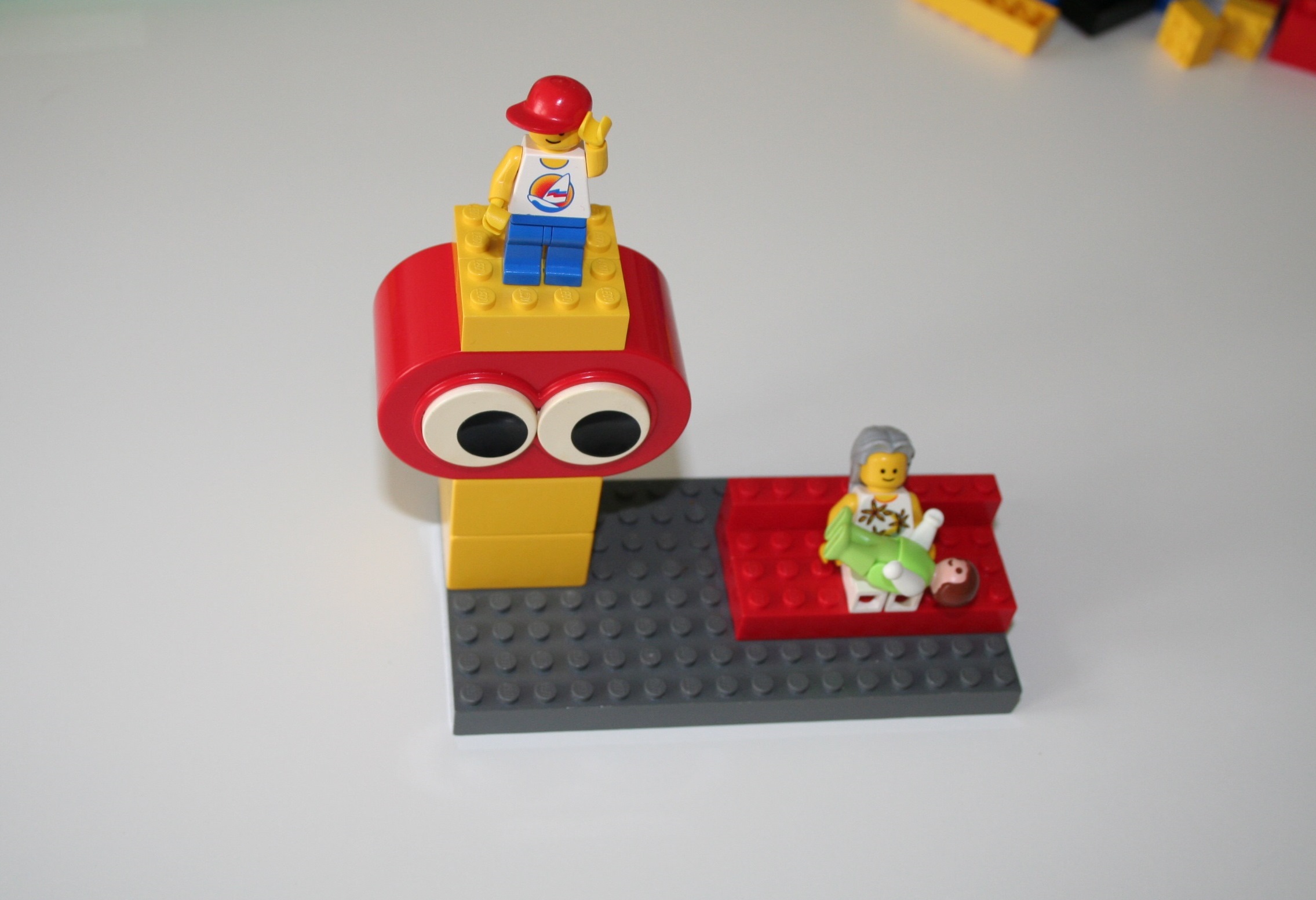

Supplement: Additional file 2: Picture S2. — Illustration of the health professional keeping a watchful eye upon the health and wellbeing of mother and child. (constructed by Aline, 29 years, first child, 1 month, cesarean). (JPG 426 kb) [file 12913_2016_1300_MOESM2_ESM.jpg]

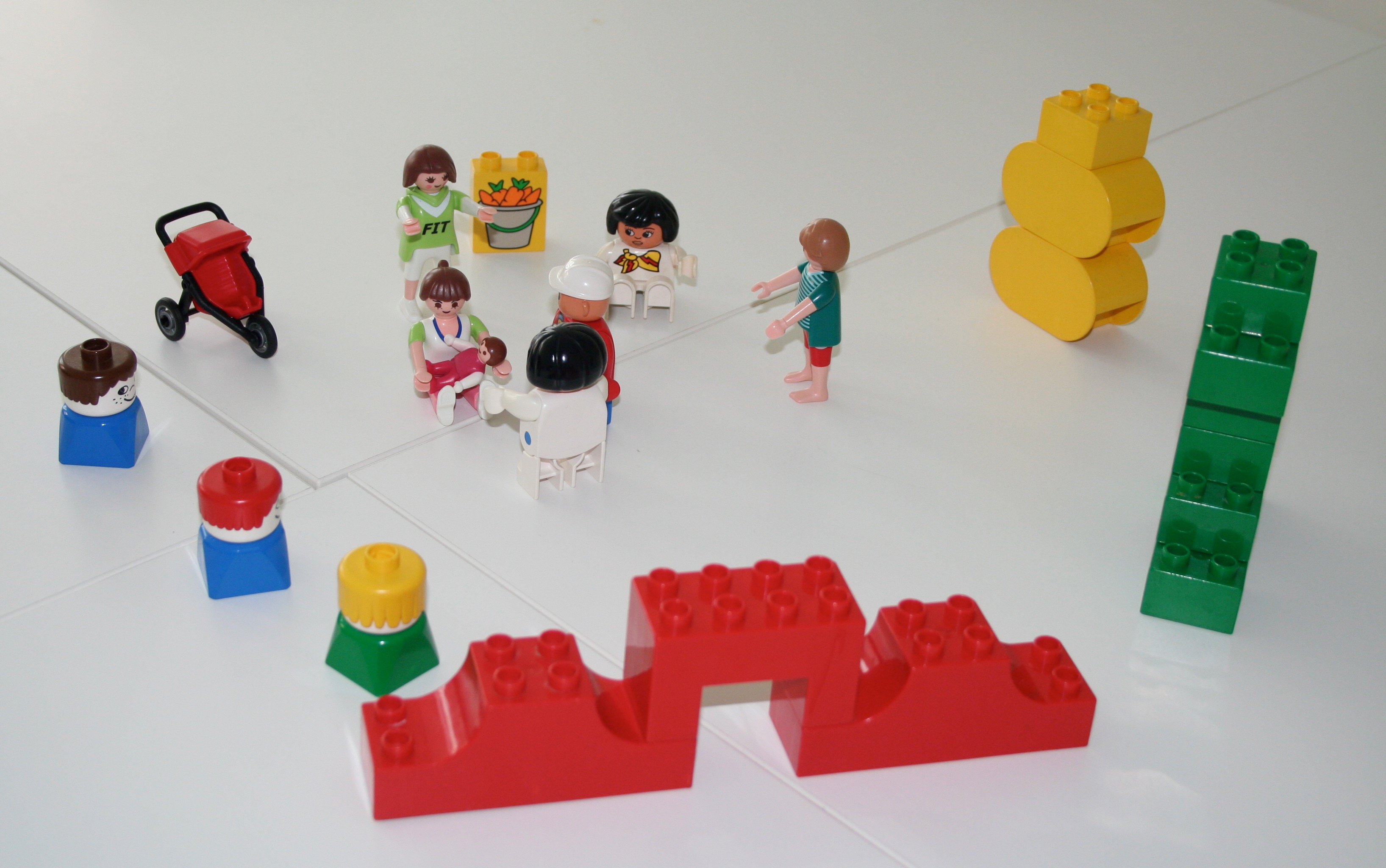

Supplement: Additional file 3: Picture S3. — Care model developed by German-speaking, first-time and multiparous women: Mother, child, father and midwife are in the center. The red bridge illustrates the role of the case manager linking the family to the other players. (JPG 1484 kb) [file 12913_2016_1300_MOESM3_ESM.jpg]

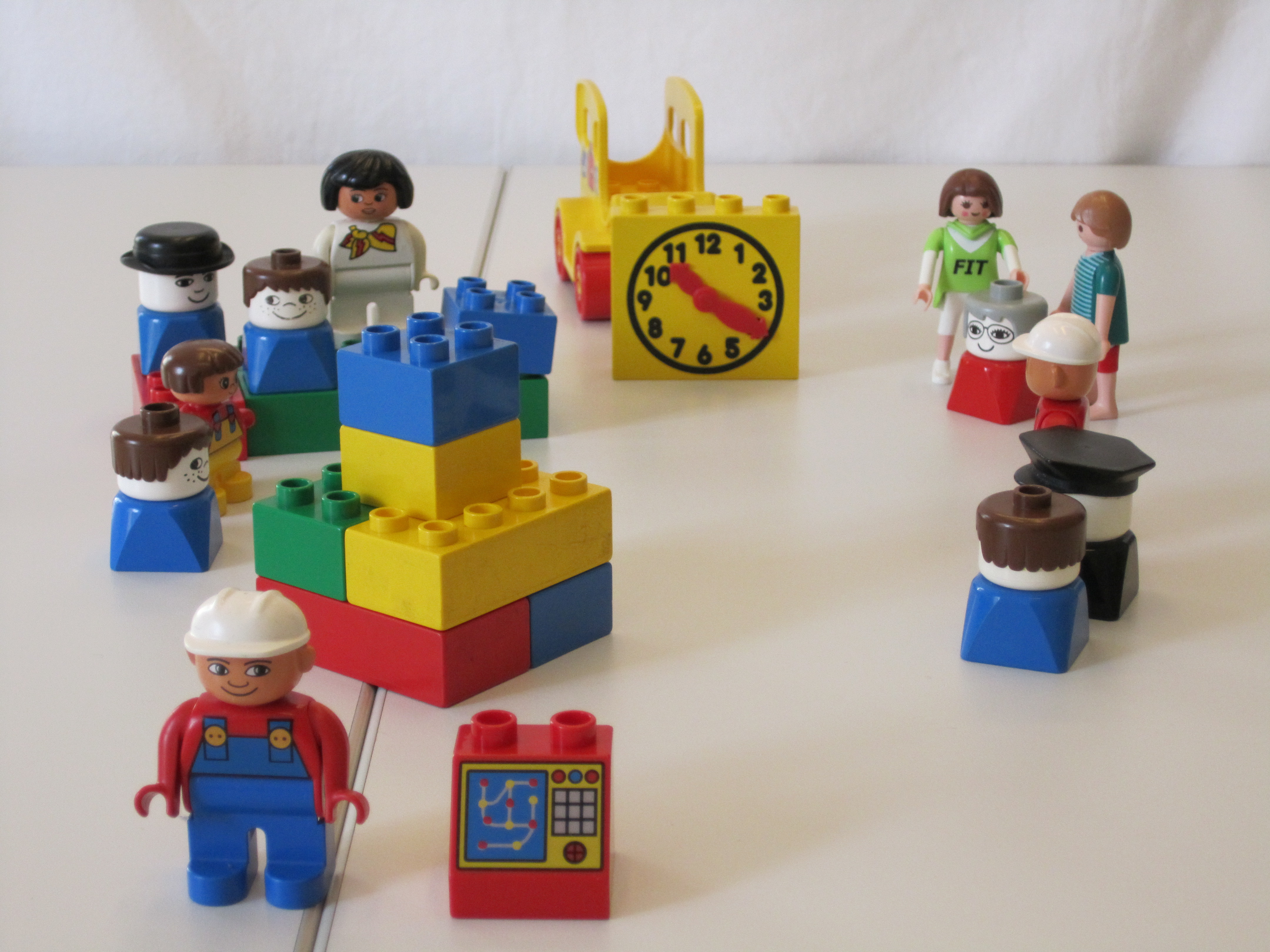

Supplement: Additional file 4: Picture S4. — Care model developed by German-speaking, first-time mothers: The family and the health professionals are arranged around the central office. The watch represents 24-hour availability and the computer in the foreground represents the telephone and bundled information available from the central office. (JPG 2982 kb) [file 12913_2016_1300_MOESM4_ESM.jpg]
